# Development and Validation of a Model to Identify Critical Brain Injuries Using Natural Language Processing of Text Computed Tomography Reports

**DOI:** 10.1001/jamanetworkopen.2022.27109

**Published:** 2022-08-16

**Authors:** Victor M. Torres-Lopez, Grace E. Rovenolt, Angelo J. Olcese, Gabriella E. Garcia, Sarah M. Chacko, Amber Robinson, Edward Gaiser, Julian Acosta, Alison L. Herman, Lindsey R. Kuohn, Megan Leary, Alexandria L. Soto, Qiang Zhang, Safoora Fatima, Guido J. Falcone, M. Seyedmehdi Payabvash, Richa Sharma, Aaron F. Struck, Kevin N. Sheth, M. Brandon Westover, Jennifer A. Kim

**Affiliations:** 1 Department of Neurology, Yale University, New Haven, Connecticut; 2 Department of Neurology, University of Wisconsin, Madison; 3 Department of Radiology, Yale University, New Haven, Connecticut; 4 William S Middleton Veterans Hospital, Madison, Wisconsin; 5 Department of Neurology, Massachusetts General Hospital, Boston

## Abstract

**Importance:**

Clinical text reports from head computed tomography (CT) represent rich, incompletely utilized information regarding acute brain injuries and neurologic outcomes. CT reports are unstructured; thus, extracting information at scale requires automated natural language processing (NLP). However, designing new NLP algorithms for each individual injury category is an unwieldy proposition. An NLP tool that summarizes all injuries in head CT reports would facilitate exploration of large data sets for clinical significance of neuroradiological findings.

**Objective:**

To automatically extract acute brain pathological data and their features from head CT reports.

**Design, Setting, and Participants:**

This diagnostic study developed a 2-part named entity recognition (NER) NLP model to extract and summarize data on acute brain injuries from head CT reports. The model, termed *BrainNERD*, extracts and summarizes detailed brain injury information for research applications. Model development included building and comparing 2 NER models using a custom dictionary of terms, including lesion type, location, size, and age, then designing a rule-based decoder using NER outputs to evaluate for the presence or absence of injury subtypes. BrainNERD was evaluated against independent test data sets of manually classified reports, including 2 external validation sets. The model was trained on head CT reports from 1152 patients generated by neuroradiologists at the Yale Acute Brain Injury Biorepository. External validation was conducted using reports from 2 outside institutions. Analyses were conducted from May 2020 to December 2021.

**Main Outcomes and Measures:**

Performance of the BrainNERD model was evaluated using precision, recall, and F1 scores based on manually labeled independent test data sets.

**Results:**

A total of 1152 patients (mean [SD] age, 67.6 [16.1] years; 586 [52%] men), were included in the training set. NER training using transformer architecture and bidirectional encoder representations from transformers was significantly faster than spaCy. For all metrics, the 10-fold cross-validation performance was 93% to 99%. The final test performance metrics for the NER test data set were 98.82% (95% CI, 98.37%-98.93%) for precision, 98.81% (95% CI, 98.46%-99.06%) for recall, and 98.81% (95% CI, 98.40%-98.94%) for the *F* score. The expert review comparison metrics were 99.06% (95% CI, 97.89%-99.13%) for precision, 98.10% (95% CI, 97.93%-98.77%) for recall, and 98.57% (95% CI, 97.78%-99.10%) for the *F* score. The decoder test set metrics were 96.06% (95% CI, 95.01%-97.16%) for precision, 96.42% (95% CI, 94.50%-97.87%) for recall, and 96.18% (95% CI, 95.151%-97.16%) for the *F* score. Performance in external institution report validation including 1053 head CR reports was greater than 96%.

**Conclusions and Relevance:**

These findings suggest that the BrainNERD model accurately extracted acute brain injury terms and their properties from head CT text reports. This freely available new tool could advance clinical research by integrating information in easily gathered head CT reports to expand knowledge of acute brain injury radiographic phenotypes.

## Key Points

**Question:**

Can a named entity recognition (NER) natural language processing and decoding model extract information about all acute brain injuries described in the text reports of computed tomography head scans?

**Findings:**

This diagnostic study including data from 1152 patients found that the NER model could extract acute pathological findings and their descriptive diagnostic properties from radiology text reports.

**Meaning:**

These findings suggest that large data sets of head radiology text reports can be quickly and accurately analyzed to advance investigations of various injuries using radiographic findings and help to characterize injuries via radiography in hospitalized patients with acute brain injuries.

## Teaser

This diagnostic study develops and validates a named entity recognition and decoder natural language processing model for extracting data from text head computed tomography reports.

## Introduction

Acute brain injuries, including ischemic strokes, intracerebral hemorrhages (ICHs), subarachnoid hemorrhages, and traumatic brain injuries (TBIs), are often associated with morbidity and mortality, with impacts on both patients and their families. More than 1 million people in the US experience acute brain injury yearly.^1,2^ Improving our ability to predict complications and long-term outcomes in acute brain injury is essential for informed medical decision-making and optimal care. Increasingly, investigators are using large multi-institutional data sets for insights.

A major obstacle to developing multi-institutional data sets is efficient and accurate data extraction and interpretation. Radiographic imaging is critical in acute brain injury. Features in these images^3,4^ can be analyzed with machine learning (ML) techniques, such as radiomics,^5,6^ image segmentation,^7,8,9^ or volumetric analyses.^10,11^ However, many of these methods are not broadly accessible and require specialized, high-resolution images and significant image processing infrastructure.^12,13^

Radiology reports, which are free-text reports by radiologists summarizing clinical imaging findings, are rich sources of information about imaging pathological characteristics. However, manual imaging report review is inefficient and subject to human error. Natural language processing (NLP) uses ML techniques to identify key findings in text and is increasingly popular in research for radiography report evaluation.^14,15,16^ Nearly every hospitalized patient with acute brain injury undergoes head computed tomography (CT), generating thousands of lines of text. As access to multicenter patient data expands, NLP may become an increasingly important means of eliciting critical data from unstructured neuroradiology reports.

Named entity recognition (NER) is an NLP technique used to extract key terms from free text.^17,18,19,20^ Words, phrases, and numbers are “entities” that can describe injury, size, and location and are amenable for analysis using ML algorithms. One example is the EdIE-R system,^21,22^ developed using artificial intelligence NER and a rule-based system based on reports from the Edinburgh Stroke Study to identify ischemic and hemorrhagic strokes. The output of this model is restricted to a small number of stroke-related entities, limiting its utility in other neurological conditions. Other attempts at using NLP techniques in neuroradiology have similarly focused on single diseases.^23,24,25^ Some studies focus on simple classification schemes of “normal” vs “abnormal” reports without further granularity, such as injury location, size, and chronicity,^26,27^ thereby limiting their practicality for research detailing injury characteristics. Combining access to a large data set of radiological reports with a large, comprehensive NER dictionary could yield an accurate and flexible NLP system capable of extracting and evaluating a broad range of acute brain injury phenotypes.^28^

We aimed to build a publicly available, novel, 2-part NLP model using NER and a rule-based decoder, which we named *BrainNERD*. We hypothesize that BrainNERD can accurately detect and extract a wide range of information on brain injuries and their characteristics, such as size or location, from head CT reports for a variety of research applications.

## Methods

BrainNERD contains 2 parts: a NER model and a rule-based decoder model. This diagnostic study is a retrospective analysis of prospectively collected data from the Yale Acute Brain Injury Biorepository and was approved by the Yale Institutional Review Board, as well as all institutions contributing data, with a waiver of informed consent because the study did not pose any risk to the patient or provide any identifiable information linking the data to the patient. This report conforms to the Standards for Reporting of Diagnostic Accuracy (STARD) reporting guideline for prediction model development and validation.

### Part I: NER Model

#### Derivation Cohort Data Set

Our derivation data set includes 3361 noncontrast head CT reports from 1152 adult patients in the Yale Longitudinal Study of Acute Brain Injury. This repository contains clinical and demographic data on more than 4000 patients hospitalized with acute brain injuries in Yale New Haven Hospital’s Neurosciences Intensive Care Unit since 2017. Patients with acute brain injuries who had head CT scans during their acute hospitalization were included.

#### External Validation Cohort Data Set

We externally validated our model using 500 head CT reports from Massachusetts General Hospital (MGH) and 553 head CT reports from the University of Wisconsin (UW). MGH reports contained a mix of admitted patients with neurologic and systemic disease. The UW cohort included patients with acute brain injuries, similar to Yale’s cohort (top 3 diagnoses: ischemic stroke, TBI, and ICH).

#### Deidentification

We deidentified all head CT reports using a regular expressions algorithm to remove all keywords and phrases with patient identifiers in the text using Python software version 3.7 (Python Software Foundation). Full deidentification was confirmed during the manual tagging process.

#### Entity Dictionary

We created a custom dictionary describing entities classified into 5 major categories: injuries, magnitude, location, time, and other. The entities and terms within each entity were defined by board-certified, acute care neurologists (G.E.G. and J.A.K.). The other category included negation, uncertainty, and end-line (ie, “.”) terms.

#### Manual Head CT Report Tagging

To assist with manually assigning each entity we used an open-source software, TagEditor,^29^ that allows users to predefine entities and tag or label dictionary terms found in the text according to their entity category. We divided data among 5 trained annotators in 2 rounds of review (a unique 20% of training data per round), to reduce mislabeling errors and identify missing dictionary terms. All data were reviewed by at least 1 neurologist (G.E.G or J.A.K.). Discrepancies were adjudicated by 1 neurologist (J.A.K.).

#### NER Models

We built 2 NER systems to deploy our NLP Model. We used spaCy version 2.3.2, an open-source Python library from Explosion and Pytorch (Meta AI), to train and deploy our first NLP model.^30^ The architecture^31^ is not open-source but has been described as akin to a hierarchical attention network for document classification.^32^ Our first NER model is based on the spaCy pipeline for tokenization, tagging, and parsing using their fixed set of hyperparameters. Given the rise of transformer architecture use,^32^ we built a second NER model using a spaCy library that integrates the bidirectional encoder representations from transformers.^33^ The bidirectional encoder representations from transformers architecture uses attention mechanisms^34^ that improve on traditional recurrent neural network using sequence-to-sequence methods^35^ by understanding language in context, which traditional sequence-to-sequence methods cannot do. We were able to compare the performance of these 2 model architectures in our data.

#### Training, Validation, and Testing Sets

**Figure 1.**
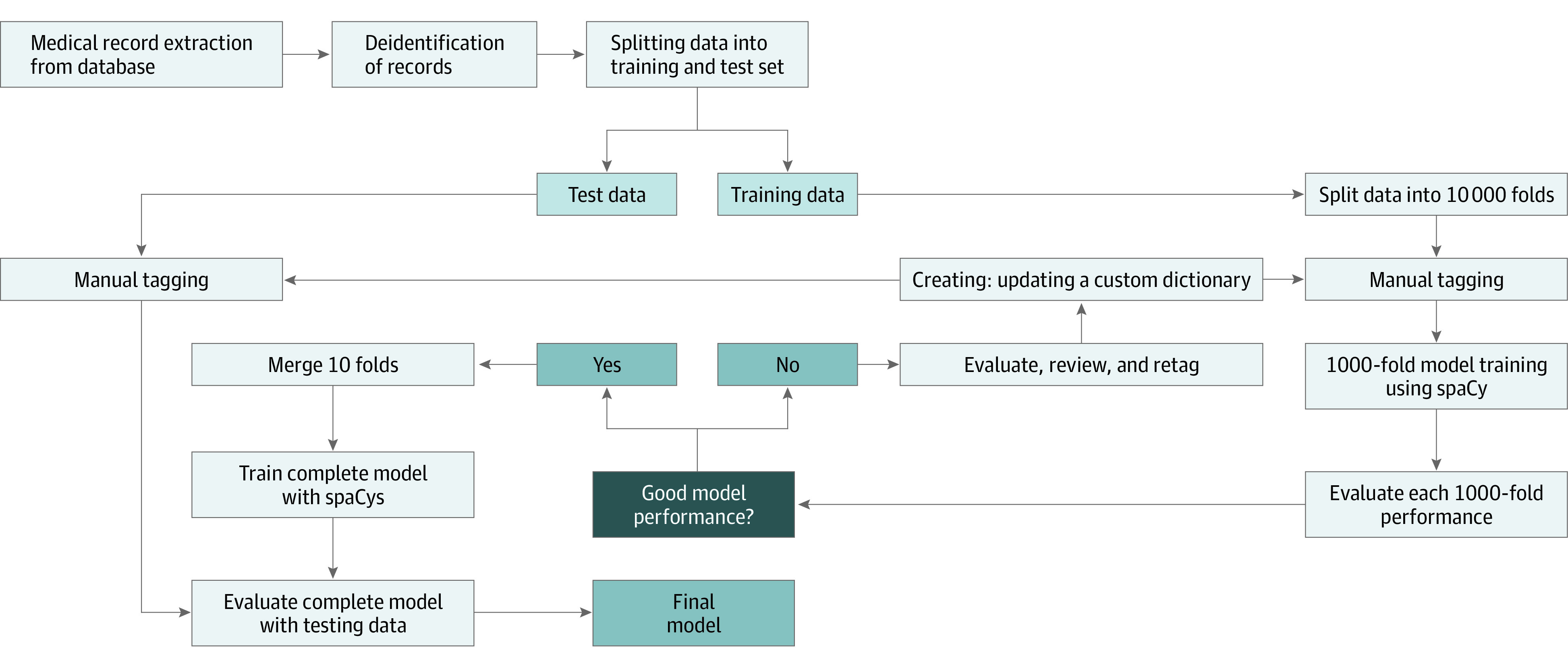
Named Entity Recognition Model Workflow

Sentence-based evaluation resulted in 46 275 sentences (including findings not indicative of injury and anomalous findings) from 3361 CT scans. We split our data set per report and patient to avoid within-person correlation into 2521 CTs with 34 706 sentences (75%) for training and 840 CTs with 11 568 sentences (25%) for testing. Our training set was split for 10-fold cross-validation for parameter optimization and interim assessment of entity performance. After cross-validation, a final model was created using the entire training data set (Figure 1). The test set was divided into 2 equal parts, with one comparing manual tagging based on our dictionary and the other used to assess our models’ performance against expert review (performed by J.A.K.).

#### Model Performance Assessment

Final model performance was assessed on our test and external data sets using precision, recall and *F* score measures. Precision was calculated as true positive / (true positive + false positive). Recall was calculated as true positive / (true positive + false negative). *F* score was calculated as 2 × (precision × recall) / (precision + recall).

#### Entity Outputs

We created custom functions to export the data in 2 matrix or table formats to provide flexibility. One format used 2 columns and was termed our *long format*. The other format used 1 entity per column and was termed our *wide format*.

### Part II: Decoder Model

#### Patient Data

We used 1 of the aforementioned 10 cross-validation folds (271 reports) to build the second part of our NLP model. The long-format data outputs from the NER model served as the input for our decoder model. The purpose of this model is to summarize the presence, absence, or possibility of each injury type within a CT report’s findings and interpretation sections. Because the classification model is rule-based, we used a smaller training data set to avoid overfitting. Thus, 75 reports (713 sentences) were randomly designated as training data, and 196 reports (1746 sentences) were designated as test data. Each report contains a large mixture of presence or absence of injuries, allowing for a robust data set to classify the presence or absence of each injury category. This is unique from other models that have only classified a report as “normal” or ”abnormal.” Our goal was to provide more detailed classifications and descriptions of injuries.

#### Decoder Terms

We chose a representative set of injury entity categories. The categories were hemorrhage, stroke, hydrocephalus, surgical intervention, herniation, mass effect, midline shift, edema, fluid, lesion, pneumocephalus, vascular malformation, and density (including high, low, mixed, and undifferentiated).

#### Decoder Labeling

To validate the accuracy of our classification model, 2 trained research associates (A.J.O. and S.M.C.) and 2 neurocritical care advanced practice clinicians (A.R. and E.G.) each labeled 50% of the data. All data was adjudicated by physicians (G.E.G. and J.A.K.). Each report was labeled according to whether an injury was positive, possibly present (eg, “likely represents a [condition]”), not present, or not mentioned. The following rules were applied: (1) if an injury is mentioned multiple times in a report, the label used was according to the most positive term, such that “positive” outweighs “possibly present,” which outweighs “not present” or “not mentioned”; (2) if an injury is mentioned as being present in the recent past (eg, evolving infarct, stable hemorrhage), then that injury is marked as positive; and (3) descriptions of chronic disease (eg, small vessel ischemic disease, atrophy) and injuries outside the skull were ignored for this model.

#### Decoder Model

**Figure 2.**
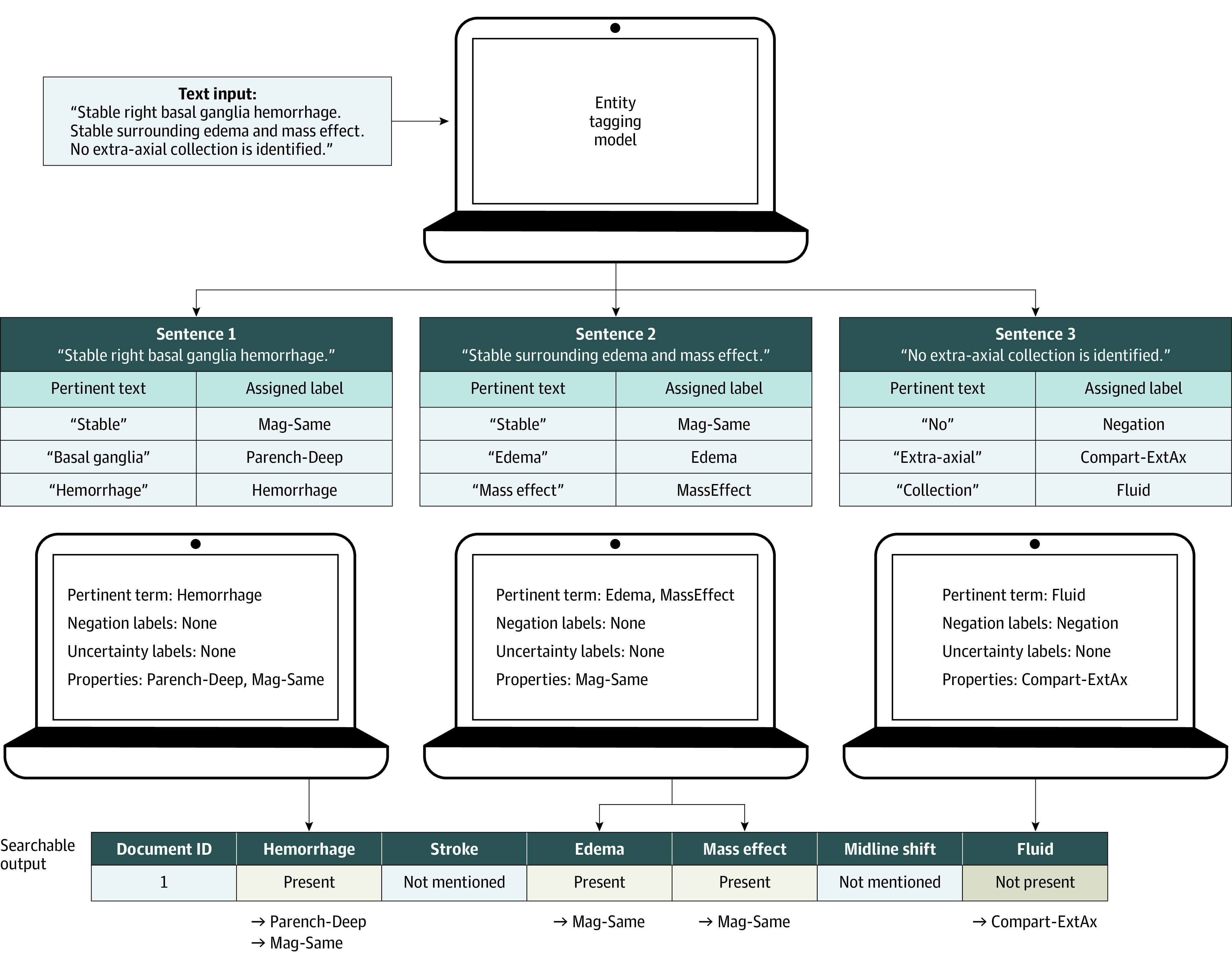
Graphic Representation of Text Input to Searchable Output Vector Step 1 of the process is to input unstructured text from a head computed tomography report; step 2, identify clinically relevant injury terms present in the text using the named entity recognition output in long-data format; step 3, identify pertinent injury, negation, uncertainty and property terms from the long data; step 4, produce an output summary of these terms.

For each report, default labels are set to not mentioned. The classification model separates the long data output from the NER model into individual sentences. It then iterates over each text-entity pair in a sentence and searches for the presence of injury entities. When the classification model detects an injury, it updates the tag for this term. If the injury term is present with no negation or uncertainty term, then the injury is tagged as positive. If the sentence contains a negation beforehand, then it is tagged as negative. If the term is mentioned with an uncertainty term only, then it is tagged as possibly present (Figure 2). The use of this 2-step approach helps overcome limitations of regular expressions.^36^

### Statistical Analysis

*F* score, precision, and recall values were used to evaluate our classification model performance based on the correct classification of each injury category. We calculated 95% CIs by bootstrapping methods (resampling: NER, 5000 sentences; decoder, 100 reports; iterations, 1000). Model performance analyses were conducted between May 2020 and December 2021. The code for this model is publicly available elsewhere.^37^ All CT data used for training and testing are available on request.

## Results

### Demographics

A total of 1152 patients (mean [SD] age 67.6 [16.1] years; 586 [52%] men) were included in the training set. Among these, primary diagnosis was ischemic stroke for 566 patients (50.4%), ICH for 366 patients (29.9%), subarachnoid hemorrhage for 112 patients (10.0%), TBI for 37 patients (3.3%), IVH for 14 patients (1.2%), and TIA for 43 patients (3.8%), plus 14 patients (1.2%) with stroke mimics included as negative samples (eTable 1 in the Supplement).

### Part I: BrainNERD

#### Entity Dictionary

**Figure 3.**
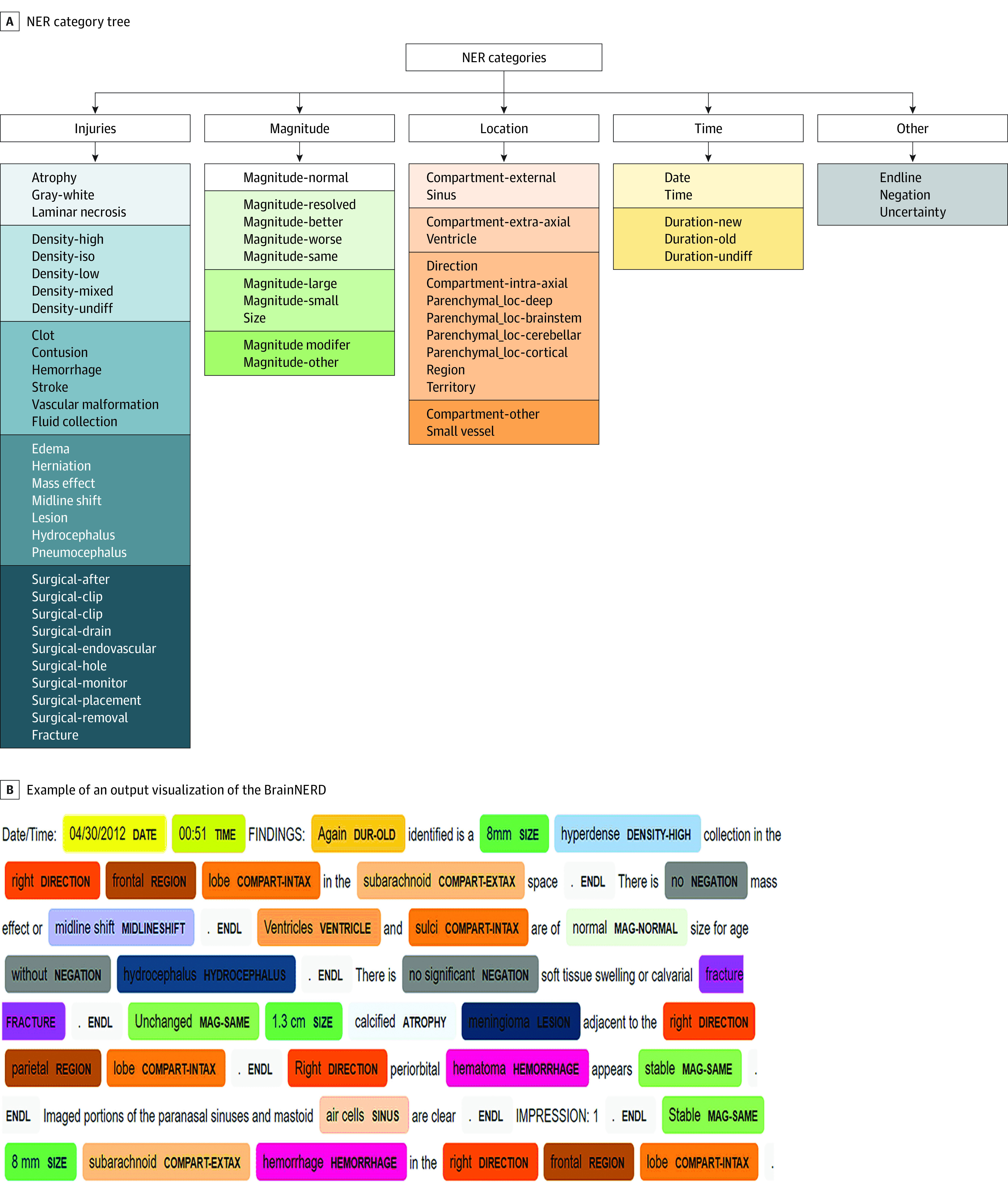
Named Entity Recognition Category Tree and Output Visualization A, The named entity recognition (NER) category tree contains all the entities in the model. The entities are divided into 5 major categories for better conceptualization and visualization of the entities. B, Example of an output visualization of the 2-part NLP model using NER and a rule-based decoder (BrainNERD) on actual head computed tomography report.

Our final custom dictionary was composed of 64 entities and 469 terms. Entities were classified into 5 major categories: injuries, magnitude, location, time, and other (Figure 3A).

#### Cross-Validation and Final Model Results

We used 10 000 cross-validations to test the model and refine terms in the entity categories. The performance for each of the 10 folds of cross-validation was 93% to 99% (Table). Our final spaCy NER model performance on half the independent test data set was 98.82% (95% CI, 98.37%-98.93%) for precision, 98.81% (95% CI, 98.46%-99.06%) for recall, and 98.81% (95% CI, 98.40%-98.94%) for *F* score. We also performed an expert review error analysis by a neurointensivist (J.A.K.) using the other half test data set, in which all injuries and their features were tagged as they would be in the clinical setting beyond the limits of the dictionary. The scores for this expert comparison were 99.06% (95% CI, 97.89%-99.13%) for precision, 98.10% (95% CI, 97.93%-98.77%) for recall, and 98.57% (95% CI, 97.78%-99.10%) for *F* score. These scores suggest accurate extraction of relevant terms within our institutional setting compared with a traditional health record review.

**Table.** NER Performance Metrics Compared With Expert Review and External Validation Data Sets

Validation	10 000 Cross validation, mean (range), %	% (95% CI)			
		Final model	Expert review	External data set	
				MGH	UW
**spaCy **					
Precision	98.09 (93.62-99.31)	98.82 (98.37-98.93)	99.06 (97.89-99.13)	98.51 (97.91-98.89)	96.31 (95.39-96.91)
Recall	98.12 (93.46-99.93)	98.81 (98.46-99.06)	98.10 (97.93-98.77)	98.40 (97.89-98.63)	96.87 (95.65-97.19)
*F* Score	98.1X (95.13-99.41)	98.81 (98.40-98.94)	98.57 (97.78-99.10)	98.95 (98.42-99.08)	96.59 (95.48-97.13)
**Transformers **					
Precision	NA	97.50 (97.49-97.52)	98.60 (98.58-98.63)	99.16 (99.14-99.19)	97.71 (97.70-97.74)
Recall	NA	99.32 (99.29-99.34)	99.10 (99.06-99.13)	98.75 (98.73-98.78)	98.70 (98.67-98.73)
*F* Score	NA	98.40 (98.39-98.43)	98.83 (98.82-98.87)	98.95 (98.91-98.96)	98.20 (97.17-99.21)

Abbreviations: MGH, Massachusetts General Hospital; NA, not applicable; NER, named entity recognition; UW, University of Wisconsin.

The transformer NER model performance on the test data set was 97.50% (95% CI, 97.49%-97.52%) for precision, 99.32% (95% CI, 99.29%-99.34%) for recall, and 98.40% (95% CI, 98.39%-98.43%) for *F* score. The scores for the transformer model expert comparison test data set were 98.60% (95% CI, 98.58%-98.63%) for precision, 99.10% (95% CI, 99.06%-99.13%) for recall, and 98.83% (95% CI, 98.82%-98.87%) for *F* score. Thus, there was no difference between NER model performances. However, there was a significant difference in model training time: the spaCy model required 40 hours (2 V100 graphics processing units [GPUs; NVIDIA]), whereas the transformer model required4 hours (1 V100 GPU). This dramatic improvement in efficiency likely relates to the underlying transformer architecture, to better use GPU parallelization.^38^ The NER performance of each entity category is reported in eTable 2 in the Supplement.

#### External Validation Results

To determine whether our models were overfit to our institution-specific terms, we tested our final model’s performance in 2 external data sets (MGH and UW; Table). The spaCy model performance for MGH data was 98.51% (95% CI, 97.91%-98.89%) for precision, 98.40% (95% CI, 97.89%-98.63%) for recall, and 98.95% (95% CI, 98.42%-99.08%) for *F* score, and the transformer model performance was 99.16% (95% CI, 99.14%-99.19%) for precision, 98.75% (95% CI, 98.73%-98.78%) for recall, and 98.95% (95% CI, 98.91%-98.96%) for *F* score. The spaCy model performance for the UW data was 96.31% (95% CI, 95.39%-96.91%) for precision, 96.87% (95% CI, 95.65%-97.19%) for recall, and 96.59% (95% CI, 95.48%-97.13%) or recall, and the transformer model performance was 97.71% (95% CI, 97.70%-97.74%) for precision, 98.70% (95% CI, 98.67%-98.73%) for recall, and 98.20% (95% CI, 97.17%-99.21%) for *F* score.

#### Outputs

The extraction of the entities and their terms in a simple format is essential for usability of BrainNERD. To visually inspect NER output in context, we used the model to automatically tag reports for review (Figure 3B). We also generated 2 output formats, long and wide, for each report (eTable 3 in the Supplement) for end-user flexibility.

### Part II: Decoder

#### Results

We evaluated the classification model against manually labeled data. For the purposes of assessing performance, not mentioned and pertinent negative categories were considered equal. The classification model performance using spaCy NER output was 96.06% (95% CI, 95.01%-97.16%) for precision, 96.42% (95% CI, 94.50%-97.87%) for recall, and 96.18% (95% CI, 95.151%-97.16%) for *F* score. Performance metrics using the transformer NER output were nearly identical, with 95.90% (95% CI, 94.28%-97.45%) for precision, 96.23% (95% CI, 94.50%-97.56%) for recall, and 96.00% (95% CI, 94.39%-97.44%) for *F* score (eTable 4 in the Supplement). External validation performance was 93.40% (95% CI, 91.41%-95.30%) for precision, 92.76% (95% CI, 90.38%-94.88%) for recall, and 92.84% (95% CI, 90.76%-94.93%) for *F* score.

#### Outputs

**Figure 4.**
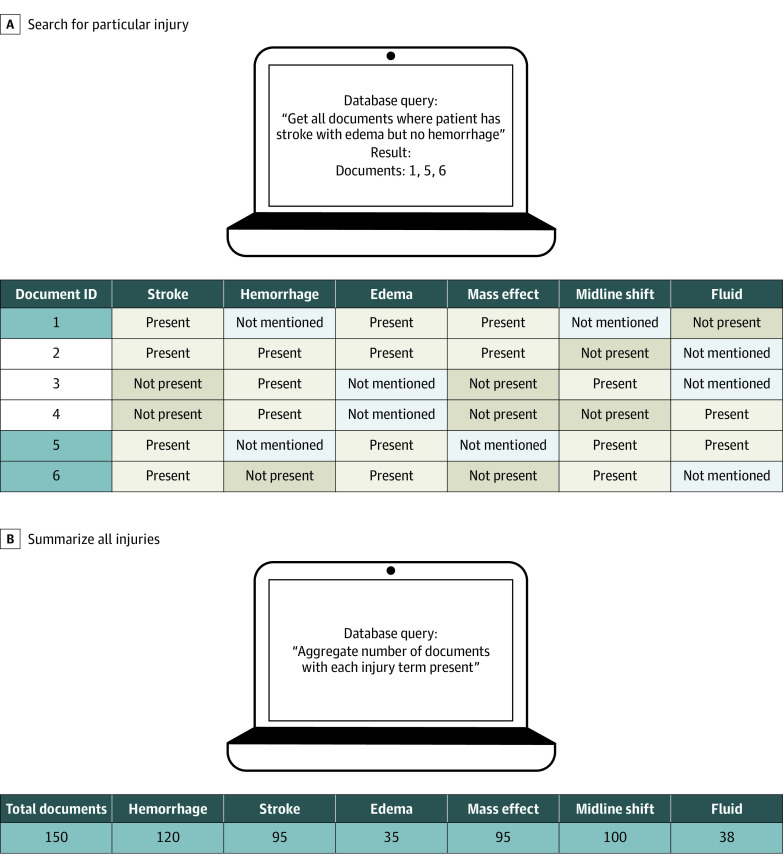
Example Applications for the BrainNERD Model Outputs

The final output of our publicly available BrainNERD model was designed for structured query language–based queries to facilitate research applications. For example, if a research team has thousands of CT reports in which they want to identify reports with a specific injury pattern, such as ischemic strokes with edema but without hemorrhage, a simple query can identify the relevant reports (Figure 4A). A second application is to summarize all the injuries found within a particular cohort of interest (Figure 4B).

## Discussion

This diagnostic study found that with more than 460 terms and modifiers present under the umbrella of 64 main entities, our BrainNERD model accurately captured all clinically relevant acute brain injury information in head CT reports, providing a broad range of future applications. The extensive dictionary allows for accurate extraction of relevant injuries and their features using novel application of readily available NER methods, followed by a decoder model which efficiently summarizes these injuries. By leveraging NLP, we eliminate the tedious, resource-intensive manual extraction previously required. To our knowledge, no prior publication has developed such a broad and detailed descriptive output of brain injuries from neuroimaging reports. Thus, researchers interested a broad range of brain injuries (and combinations thereof) will be able to flexibly use our BrainNERD tool, rather than being limited to a small subset of injuries. Our results suggest BrainNERD that can be used to robustly integrate CT report text into future research efforts.

Unlike most prior NLP efforts involving neuroradiology scans focused on identifying single or few injuries (eg, ischemic stroke),^22,23,25,26,27,38^ our goal was to create an NLP system that could be broadly applied to characterize all forms of acute brain injuries. We believe that the main advantage of BrainNERD is derived from our large dictionary of terms that describe acute brain injury features on head CT. This large dictionary enables our model to extract many diverse, granular, and high-level injury features that may be missed without expert input.

While the NER output (part I) can be used directly for research exploration, we sought to make the output even more accessible by creating a decoder model that can query for injuries of interest. For example, using BrainNERD, we can distinguish between the certainty of an injury’s presence (present vs possible) or absence. We can also extract associated properties describing the injury, including size, location, and chronicity. For example, our system can recognize the following hypothetical entities in a report sentence: “acute,” “right-sided,” “5 mm,” “subdural,” and “hemorrhage.”

Given the specificity and range of detectable phenotypes and modifiers, BrainNERD can be used for multiple applications. As previously mentioned, it can be used to search and filter for specific for different injury phenotypes, like size or location. Once identified, these patients can compose a new cohort for analysis. Alternatively, researchers can export and tally all injury information in a predefined cohort of patients. BrainNERD outputs can also be used to automatically generate common imaging scores (eg, Marshall Score, Rotterdam Score, modified Fisher scale). Thus, BrainNERD outputs provide multiple options for the robust use of information contained in neuroradiology reports. BrainNERD can be used when raw imaging data analysis is unavailable or to supplement imaging models.

There are 2 major NLP libraries capable of deep learning detection and extraction of phenotypic information to process the language of a radiology report: spaCy and the Natural Language Toolkit (NLTK).^39^ We chose to use spaCy based on some perceived advantages.^40^ One of the main differences between these libraries is that spaCy uses a word2vector model and NLTK does not. Additionally, the spaCy library is more easily deployable, by providing out-of-the-box pipelines, formulas, and code.^41^ Lastly, spaCy has faster performance in many areas compared with NLTK.^42^

### Limitations

This study has some limitations. While our model has multiple advantages over prior efforts, there are a few notable limitations. We developed a large dictionary of terms based on one of the largest data sets reported for building an NLP model of neuroradiology reports. All training data reports originated from 1 institution. To circumvent potential variance in some of the terms used at different institutions we tested 2 external validation test sets with high performance. Ultimately, a large, multi-institutional collaboration, including reports from outpatients, would allow us to fully test the flexibility of our system. The second limitation of our study is that we used a supervised, dictionary-based form of ML and NER. We did not explore alternative, unsupervised, or semisupervised methods for comparison. The creation of this large dictionary itself was time-consuming and required intense input from neurologists and researchers to optimize the organization and capture of potential terms. We used this approach to create highly detailed and specific results relevant to acute brain injuries. Overall, the effort of creating the dictionary was small compared with the effort needed to collate large cohorts for projects through manual health record review. Now that we have extensively developed and tested our model, we hope it can easily be used by researchers for future analysis and decrease future effort. Third, while the classification model (part II) enhances our ability to summarize the data output from the NER (part I), our classification model is dependent on the NER output, so any errors are propagated. In the future, we hope to expand our model to other forms of radiology reports, such as CT angiogram and magnetic resonance imaging reports, to broaden the scope of neuroradiologic findings, using BrainNERD as a prototype.

## Conclusions

The findings of this diagnostic study suggest that BrainNERD provides a new, publicly available tool allowing researchers to leverage the rich text information contained in head CT reports. By identifying a broad range of brain injuries, BrainNERD is the first system, to our knowledge, that can be used to test a wide variety of hypotheses in head CT text reports. This model’s automated extraction significantly enhances the feasibility of investigating radiographic markers in large multi-institutional studies in a rapid and resource-efficient manner.

## Notes

Supplement. **eTable 1.** Demographic Characteristics of Patients With Head CT Reports in the Yale Acute Brain Biorepository **eTable 2.** Data Dictionary Individual Entity Category Performances **eTable 3.** Wide- and Long-Format NER Model Output **eTable 4.** Decoder Performance Confusion Matrix
