# Error in Author Name

**DOI:** 10.1001/jamanetworkopen.2022.37810

**Published:** 2022-09-29

In the Original Investigation titled “Development and Validation of a Model to Identify Critical Brain Injuries Using Natural Language Processing of Text Computed Tomography Reports,”^1^ published August 16, 2022, there was an error in Dr Payabvash’s name in the byline. This should have appeared as Seyedmehdi Payabvash, MD. This article has been corrected.^1^
