# Supplementary material for: Development and Validation of a Model to Identify Critical Brain Injuries Using Natural Language Processing of Text Computed Tomography Reports
Source: JAMA Netw Open. 2022 Aug 16;5(8):e2227109. doi: 10.1001/jamanetworkopen.2022.27109 (PMC9382443; doi:10.1001/jamanetworkopen.2022.27109)
Supplement: Supplement. — eTable 1. Demographic Characteristics of Patients With Head CT Reports in the Yale Acute Brain Biorepository eTable 2. Data Dictionary Individual Entity Category Performances eTable 3. Wide- and Long-Format NER Model Output eTable 4. Decoder Performance Confusion Matrix [file jamanetwopen-e2227109-s001.pdf]

Supplemental Online Content

Torres-Lopez VM, Rovenolt GE, Olcese AJ, et al. Development and validation of a model to identify critical brain injuries using natural language processing of text computed tomography reports. *JAMA Netw Open.* 2022;5(8):e2227109. doi:10.1001/jamanetworkopen.2022.27109

eTable 1. Demographic Characteristics of Patients With Head CT Reports in the Yale Acute Brain Biorepository

eTable 2. Data Dictionary Individual Entity Category Performances

eTable 3. Wide- and Long-Format NER Model Output

eTable 4. Decoder Performance Confusion Matrix

This supplemental material has been provided by the authors to give readers additional information about their work.

© 2022 Torres-Lopez VM et al. *JAMA Network Open.*

eTable 1. Demographic Characteristics of Patients With Head CT Reports in the Yale Acute Brain Biorepository

	N	
**Patients**	1152	
**Age (mean (SD))**	67.6 (16.1)	
**Sex (%)**		
Male	586 (52%)	
Female	537 (48.0 %)	
**Ethnicity (%)**		
Non- Hispanic	1000 (92.3%)	
Hispanic	75 (6.9%)	
**Race (%)**		
White	796 (73.7%)	
Black or African American	164 (15.2%)	
Asian	28 (2.6%)	
Pacific Islander	0 (0.0%)	
American Indian/ Alaskan Native	0 (0.0%)	
Other	55 (5.1%)	
Unknown	37 (3.4%)	
**Diagnosis (%)**	**Yale**	**UWisc**
AIS	566 (50.4%)	48 (8.51%)
ICH	366 (29.9%)	29 (5.14%)
SAH	112 (10.0%)	26 (4.61%)
TIA	43 (3.8%)	0
TBI	37 (3.3%)	90 (15.96%)
IVH	14 (1.2%)	1 (0.18%)
OTH	14 (1.2%)	370 (65.60%)

© 2022 Torres-Lopez VM et al. *JAMA Network Open.*

eTable 2. Data Dictionary Individual Entity Category Performances

Model	spaCy			Transformer		
**Entity**	**Precision**	**Recall**	**F-score**	**Precision**	**Recall**	**F-score**
Atrophy	100	99.91	99.95	98.82	99.52	99.17
Clot	100	100	100	100	100	100
Compart-ExtAx	99.58	100	99.79	98.20	99.46	98.83
Compart-External	100	100	100	97.01	100	98.48
Compart-IntAx	99.95	99.92	99.93	99.37	99.46	99.42
Compart-Other	99.94	100	99.97	99.56	99.78	99.67
Contusion	100	100	100	100	100	100
Date	99.98	99.95	99.96	99.43	99.29	99.36
Density-High	100	100	100	96.40	100	98.17
Density-Iso	100	100	100	100	100	100
Density-Low	100	100	100	98.39	99.73	99.06
Density-Mixed	100	100	100	87.50	87.50	87.50
Density-Undiff	96.51	31.32	47.29	63.04	95.08	75.82
Direction	99.85	99.65	99.75	98.24	99.39	98.81
Dur-New	97.83	100	98.90	98.03	99.44	98.74
Dur-Old	99.59	95.66	97.59	98.38	98.95	98.66
Dur-Undiff	88.06	99.93	93.62	97.51	94.90	96.19
Edema	100	100	100	100	100	100
Endl	100	99.99	99.99	94.60	99.99	97.22
Fluid	98.38	100	99.18	98.38	98.37	98.38
Fracture	100	100	100	100	100	100
Gray-White	99.93	99.93	99.93	87.5	98.64	92.74
Hemorrhage	99.99	100	99.99	99.80	100	99.90
Herniation	100	100	100	100	99.19	99.59
Hydrocephalus	99.72	99.91	99.81	94.92	98.42	96.64
Lam_Necrosis	100	77.78	87.5	0	0	0
Lesion	100	96.49	98.21	99.07	99.07	99.07
Mag-Better	100	99.83	99.92	97.33	100	98.65
Mag-Large	99.45	99.89	99.68	97.27	98.166	97.72
Mag-Modifer	99.76	97.70	98.72	99.77	99.32	99.54
Mag-Normal	95.13	96.84	95.98	99.24	100	99.62
Mag-Other	98.53	99.62	99.07	99.09	98.65	98.88
Mag-Resolved	98.46	100	99.22	100	100	100
Mag-Same	97.62	100	98.79	99.43	97.47	98.44
Mag-Small	98.81	99.68	99.24	99.38	99.07	99.23
Mag-Worse	100	99.95	99.98	97.65	99.15	98.40
MassEffect	98.88	100	99.44	99.05	99.52	99.29
MidlineShift	99.95	100	99.98	98.79	98.79	98.79
Negation	99.97	99.73	99.85	100	99.93	99.97

© 2022 Torres-Lopez VM et al. *JAMA Network Open.*

Parench-Brainstem	100	100	100	93.65	100	96.72
Parench-Cerebellar	99.88	99.65	99.77	97.35	100	98.66
Parench-Cortical	100	99.75	99.88	98.58	98.58	98.58
Parench-Deep	100	100	100	99.33	100	99.66
Pneumocephalus	100	97.93	98.96	91.80	98.25	94.92
Region	99.96	99.86	99.91	98.87	99.87	99.37
Sinus	99.44	100	99.72	99.79	99.17	99.48
Size	99.61	93.33	96.37	94.66	89.87	92.20
SmallVessel	100	100	100	100	100	100
Stroke	94.63	94.63	94.63	98.58	98.59	98.58
Surgical-After	99.64	89.87	94.50	89.47	100	94.44
Surgical-Clip	100	100	100	100	100	100
Surgical-Drain	99.18	99.43	99.30	87.95	85.88	86.90
Surgical-Endo	96.36	95.83	96.09	88.46	95.83	92
Surgical-Hole	99.89	99.87	99.87	93.88	100	96.84
Surgical-Monitor	100	100	100	100	100	100
Surgical-Placement	92.45	100	96.08	100	93.75	96.77
Surgical-Removal	100	100	100	100	100	100
Territory	99.23	99.67	99.42	99.37	98.59	98.98
Time	87.72	99.76	93.36	94.95	98.04	96.47
Uncertainty	96.69	99.96	98.30	97.15	98.84	97.99
VascMalform	100	100	100	99.31	100	99.65
Ventricle	99.40	99.41	99.41	98.10	98.53	98.31

© 2022 Torres-Lopez VM et al. *JAMA Network Open.*

eTable 3. Wide- and Long-Format NER Model Output

		Entities	Val					
a.	0	Size	1 cm					
	1	Compartment	epidural					
	2	Hematoma	hematoma					
	3	Loc	right					
	4	Loc	anterior					
	5	Region	temporal					
	6	Magnitude	stable					
	7	Endl	.					
b.	**Size**	**Compartment**	**Hematoma**	**Loc**	**Loc**	**Region**	**Magnitude**	**Endl**
	1 cm	epidural	hematoma	right	anterior	temporal	stable	.

© 2022 Torres-Lopez VM et al. *JAMA Network Open.*

eTable 4. Decoder Performance Confusion Matrix

	True positive	True possible	True negative
spaCy			
Predicted positive	654	3	36
Predicted possible	7	8	3
Predicted negative	23	26	1976
Transformer			
Predicted positive	649	3	35
Predicted possible	7	8	3
Predicted negative	28	26	1977

© 2022 Torres-Lopez VM et al. *JAMA Network Open.*
